# Structure, Function, and Regulation of the Blood-Brain Barrier Tight Junction in Central Nervous System Disorders

**DOI:** 10.3389/fphys.2020.00914

**Published:** 2020-08-06

**Authors:** Jeffrey J. Lochhead, Junzhi Yang, Patrick T. Ronaldson, Thomas P. Davis

**Affiliations:** Department of Medical Pharmacology and Pharmacology/Toxicology, Colleges of Medicine and Pharmacy, University of Arizona, Tucson, AZ, United States

**Keywords:** blood-brain barrier, claudins, occludin, tight junctions, paracellular permeability, drug delivery

## Abstract

The blood-brain barrier (BBB) allows the brain to selectively import nutrients and energy critical to neuronal function while simultaneously excluding neurotoxic substances from the peripheral circulation. In contrast to the highly permeable vasculature present in most organs that reside outside of the central nervous system (CNS), the BBB exhibits a high transendothelial electrical resistance (TEER) along with a low rate of transcytosis and greatly restricted paracellular permeability. The property of low paracellular permeability is controlled by tight junction (TJ) protein complexes that seal the paracellular route between apposing brain microvascular endothelial cells. Although tight junction protein complexes are principal contributors to physical barrier properties, they are not static in nature. Rather, tight junction protein complexes are highly dynamic structures, where expression and/or localization of individual constituent proteins can be modified in response to pathophysiological stressors. These stressors induce modifications to tight junction protein complexes that involve *de novo* synthesis of new protein or discrete trafficking mechanisms. Such responsiveness of BBB tight junctions to diseases indicates that these protein complexes are critical for maintenance of CNS homeostasis. In fulfillment of this vital role, BBB tight junctions are also a major obstacle to therapeutic drug delivery to the brain. There is an opportunity to overcome this substantial obstacle and optimize neuropharmacology *via* acquisition of a detailed understanding of BBB tight junction structure, function, and regulation. In this review, we discuss physiological characteristics of tight junction protein complexes and how these properties regulate delivery of therapeutics to the CNS for treatment of neurological diseases. Specifically, we will discuss modulation of tight junction structure, function, and regulation both in the context of disease states and in the setting of pharmacotherapy. In particular, we will highlight how these properties can be potentially manipulated at the molecular level to increase CNS drug levels *via* paracellular transport to the brain.

## Introduction

**Figure 1:**
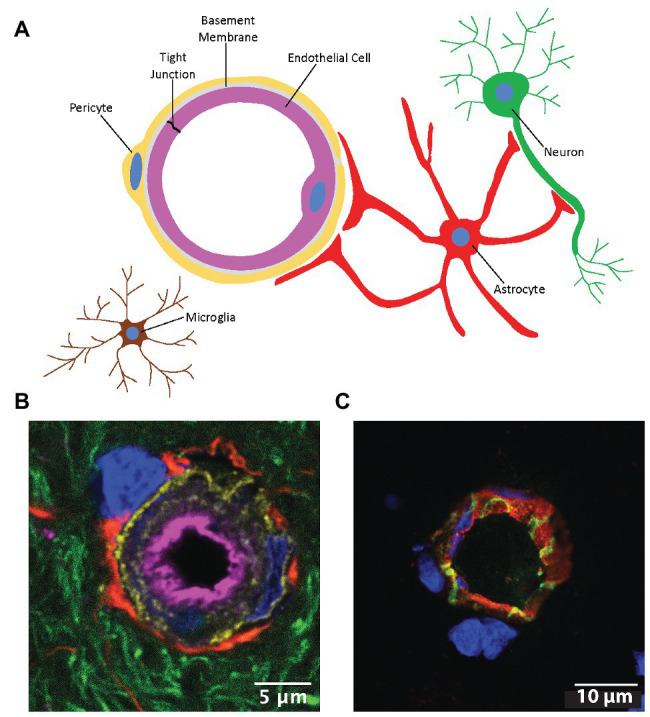
The neurovascular unit (NVU). **(A)** Cells comprising the NVU. **(B)** Confocal micrograph of a cerebral capillary labeled with lectin (magenta), pericytes labeled with an antibody to platelet derived growth factor beta (gold), astrocyte end feet labeled with glial fibrillary acidic protein (red), neurites labeled with Neuro-Chrom antibody (green), and nuclei labeled with 4',6-diamidino-2-phenylindole (DAPI; blue). **(C)** Cross-section of a microvessel labeled with lectin (red), an antibody to the tight junction (TJ) protein claudin-5 (green), and DAPI (blue). Modified with permission from Tome et al. (2018).

The neurovascular unit (NVU) is an anatomical and functional unit that responds to the needs of neuronal energy demands by precisely regulating cerebral blood flow (Iadecola, 2017). The NVU is comprised of brain endothelial cells, astrocytes, mural cells (i.e., vascular smooth muscle cells and pericytes), microglia, neurons, and extracellular matrix components (Figure 1). The microvascular endothelial cells of the NVU comprise the blood-brain barrier (BBB), a physical and metabolic barrier important for maintaining central nervous system (CNS) homeostasis and protecting the brain from potentially harmful circulating substances. The metabolic component of the BBB consists of drug metabolizing enzymes that are capable of inactivating therapeutics and/or altering their ability to enter the CNS (Agundez et al., 2014). The BBB exhibits low permeability compared to peripheral blood vessels and only lipophilic molecules with molecular weights less than 500 Da, and few hydrogen bond donors and/or acceptors are able to passively diffuse across the BBB under physiological conditions (Banks, 2009; Banks and Greig, 2019). Nutrients such as glucose, amino acids, nucleosides, and certain neurotransmitters that are important for CNS function are able to enter the brain through carrier-mediated transport (Ohtsuki and Terasaki, 2007). Influx and efflux transporters, which extrude toxic metabolites from endothelial cells and limit CNS entry of xenobiotics and therapeutics from the bloodstream, are also a prominent feature of the BBB (Abdullahi et al., 2017). While receptors and transporters expressed at the endothelial plasma membrane selectively regulate brain entry of various solutes from the blood, the major factors limiting BBB permeability are its low rate of pinocytosis and tight junction (TJ) protein complexes which limit passage through the transcellular and paracellular routes, respectively (Reese and Karnovsky, 1967).

TJs at the BBB are protein complexes that form rows of extensive, overlapping occlusions between brain microvascular endothelial cells that greatly restrict paracellular diffusion of polar molecules and macromolecules into the CNS (Reese and Karnovsky, 1967; Brightman and Reese, 1969). The *in vivo* transendothelial electrical resistance (TEER) of brain microvessels is 1,500–2,000 Ω cm^2^, which is considerably higher than peripheral microvessels that have a TEER of 3–30 Ω cm^2^ (Crone and Olesen, 1982; Butt et al., 1990). Although previously considered to be static structures, the past few decades of research have revealed that TJs at the BBB are highly dynamic in nature and are dysregulated in many CNS disease states (Erdo et al., 2017; Reinhold and Rittner, 2017; Costea et al., 2019; Sweeney et al., 2019). While TJ dysregulation at the BBB can contribute to the pathogenesis of certain neurological disorders, understanding the mechanisms involved may provide an opportunity to improve CNS drug delivery through the paracellular pathway. Here, we provide an overview of the molecular composition of TJs at the BBB, disruption of TJs during CNS diseases, and strategies to manipulate TJs with the goal of improved drug delivery to the brain.

## Molecular Composition and Regulation of Tjs at the BBB

**Figure 2:**
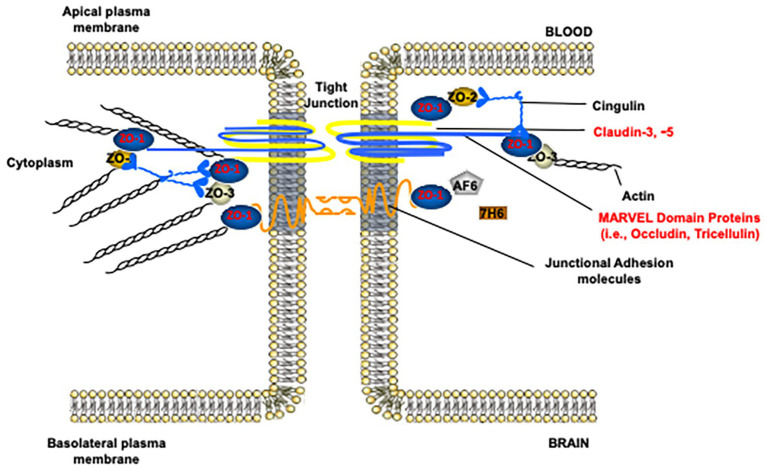
Molecular composition of tight junction protein complexes. Modified with permission from Abdullahi et al. (2018).

In the paracellular cleft, TJs form a physical seal between adjacent brain microvascular endothelial cells (Figure 2). In conjunction with active efflux transporters and the lack of fenestrations along the apical surface of the brain microvessel, TJ protein complexes contribute to physiological functioning of the BBB, including maintenance of a stable microenvironment within the CNS. TJ functional integrity at the BBB can be evaluated by measurement of extravasation of solutes (i.e., leak) that are not transport substrates and typically remain in the vascular lumen under physiological conditions. Such vascular markers cover a wide range of molecular weights and, if used appropriately, can enable a detailed evaluation of size selectivity of vascular “leak” in the setting of pathological stressors. Commonly used tracer molecules for the study of paracellular diffusion are presented in Table 1. Using transmission electron microscopy, BBB TJ protein complexes are visualized as a series of electron dense regions (i.e., “kissing points”) between apposing membranes of adjacent endothelial cells (Chiba et al., 2008). At the molecular level, TJs are comprised of transmembrane protein complexes protruding from neighboring cells. These transmembrane TJ proteins are linked to the cytoskeleton by intracellular TJ proteins, thereby forming complex networks that are highly responsive to pathological stimuli. Indeed, many different protein constituents are reported to be present at TJs in mammalian cerebral microvasculature (Haseloff et al., 2015; Reinhold and Rittner, 2017; Berndt et al., 2019). Below, we review the specific transmembrane and intracellular proteins that have a confirmed role in maintaining TJ functional integrity in health and disease.

**Table 1:** Common blood-brain barrier (BBB) permeability markers used for measurement of paracellular leak.

Tracer	Molecular weight (kDa)	Comments	References
Sucrose	0.3423	Vascular marker that does not transport and does not cross the intact BBB. Useful for measurement of transient or subtle BBB leak. Typically radiolabeled to allow for detection in brain tissue or brain fluids. Vascular compartment washout or capillary depletion is critical to prevent overestimation of brain sucrose levels.	(Bhattacharjee et al., 2001; Saunders et al., 2015)
Sodium fluorescein (NaF)	0.3763	Small molecular weight marker commonly used to assess BBB paracellular permeability. Weakly binds to plasma proteins. Transport substrate for organic anion transporter 3 (OAT3) and multidrug resistance protein 2 (MRP2). Since both proteins transport substrates from brain to blood, use of NaF may lead to underestimations in BBB leak.	(Wolman et al., 1981; Hawkins and Egleton, 2006; Kaya and Ahishali, 2011; Saunders et al., 2015)
Lucifer yellow	0.4423	Small molecular weight marker for assessment of BBB integrity. Can only access brain tissue *via* passive paracellular diffusion. Useful for measurement of transient or subtle BBB leak.	(Omidi et al., 2003; Qosa et al., 2016)
Evans blue	0.9608	Small molecular weight dye that is commonly used to assess BBB dysfunction in *in vivo* model system. Depending on the concentration used, a percentage will bind to plasma proteins such as albumin, and a percentage will remain free. Due to perceived binding to albumin, Evan’s blue staining in brain tissue is believed to reflect large-scale BBB opening; however, the fact that some dye remains unbound to plasma proteins makes it impossible to use Evan’s blue as a reflection of size selectivity of BBB leak.	(Kaya and Ahishali, 2011; Saunders et al., 2015)
Dextrans	3-2000	Available across a wide range of molecular weights, which enables assessment of molecular weight range of BBB leak. Can be conjugated to fluorescent labels [i.e., fluorescein isothiocyanate (FITC), tetramethylrhodamine] or to biotin to enable detection in brain tissue or brain fluids. Must be used at low concentrations to avoid *in vivo* toxicity.	(Willis et al., 2010; Saunders et al., 2015; Natarajan et al., 2017; Devraj et al., 2018)
Horseradish peroxidase (HRP)	44	Allows for assessment of BBB permeability to macromolecules. Reaction product can be made electron-dense, thereby enabling visualization by electron microscopy. Can cause mast cell degranulation, which can affect BBB permeability in *in vivo* experiments. Therefore, HRP may overestimate BBB leak under these conditions.	(Kaya and Ahishali, 2011; Saunders et al., 2015)
Albumin	66.5	Plasma protein that does not cross the intact BBB. Useful for evaluated large-scale BBB disruption. Can be fluorescently labeled to allow for measurement of duration or progression of BBB leak.	(Saunders et al., 2015)
Immunoglobulin G (IgG)	150	Large molecular weight tracer that enables assessment of large-scale BBB disruption. Can be fluorescently labeled to allow for measurement of duration or progression of BBB leak	(Saunders et al., 2015)
Fibrinogen	340	Large molecular weight tracer that enables assessment of large-scale BBB disruption. Can be fluorescently labeled to allow for measurement of duration or progression of BBB leak	(Saunders et al., 2015)

Claudins are considered to be the principal proteins that establish the backbone of TJ and are critical determinants of paracellular “tightness” between adjacent BBB endothelial cells (Gunzel and Yu, 2013). The claudin family of proteins consists of 27 members that have been identified to date, and family members have an approximate molecular mass of ~26 kDa (Daneman et al., 2010; Mineta et al., 2011). They have a tissue-specific expression pattern (Hwang et al., 2014; Markov et al., 2015), which leads to different barrier properties in different tissues. In epithelial cells, claudin-3 and claudin-5 generally provide a sealing function (Amasheh et al., 2005; Milatz et al., 2010). At the BBB, claudin-5 has long been recognized as the dominant TJ protein (Greene et al., 2019). Depletion of claudin-5 in transgenic mice results in morphologically normal TJs at the BBB but also leads to detrimental brain defects and early neonatal death ~10 h after birth. Furthermore, brain microvasculature of claudin-5 knockout mice is highly permeable to molecules less than 800 Da (Nitta et al., 2003). In contrast, overexpression of claudin-5 in cultured brain microvessel endothelial cells results in elevated paracellular tightness, suggesting that expression levels of claudin-5 directly correlate with TJ function (Ohtsuki et al., 2007). Such notions have recently been challenged by the study of Schlingmann et al. (2016), which suggests that changes in TJ function are not simply the result of increased or decreased protein levels but rather arises from disruption in the overall balance of all TJ proteins (Schlingmann et al., 2016). Similar to claudin-5, expression of claudin-3 has also been reported in brain microvasculature (Ohtsuki et al., 2008; Ronaldson et al., 2009), where it may play a role in the physiological sealing function of the TJ (Winkler et al., 2020). Claudin-1 has been detected at the BBB at the messenger RNA (mRNA) level (Winkler et al., 2020); however, its expression at the BBB remains controversial in part due to possible antibody cross-reactivity between claudin-1 and claudin-3 (Bauer et al., 2014). Claudin-11 and claudin-12 have also been detected at the BBB, but claudin-12 is likely not important for barrier function (Castro Dias et al., 2019; Uchida et al., 2019).

Structurally, the claudins are tetraspan proteins with two extracellular loops (ECLs) and two cytoplasmic domains that consist of a short amino (N)-terminal sequence and a long carboxyl (C)-terminal sequence. The ECLs of claudins contribute to the formation of the TJ backbones by homo‐ or hetero-dimerization or oligomerization with neighboring claudins on the same membrane (cis-interaction) and/or with those on the apposing cell (trans-interaction; Piontek et al., 2008; Piehl et al., 2010). The ECLs are also responsible for determining charge‐ and size selectivity of the claudins (Piehl et al., 2010; Piontek et al., 2011). Therefore, interactions between claudin-5 with other claudins or with other transmembrane proteins may be necessary to properly restrict paracellular diffusion of molecules of varying charges and sizes. The C-terminus of claudins exhibits great diversity among members of the claudin family and ends with a PDZ binding motif for most claudins with the exception being claudin-12 (Piontek et al., 2011; Van Itallie and Anderson, 2018). PDZ-dependent interactions at the TJ play a prominent role in the organization and modulation of TJ proteins (Itoh et al., 1999; Poliak et al., 2002). The PDZ domain on the C-terminal tail of most claudins recruits cytoplasmic proteins, particularly zonula occludens (ZOs) proteins. Additionally, PDZ domains on the C-terminal of a claudin molecule can also promote interactions with other transmembrane TJ proteins including occludin and junction adhesion molecules (JAMs; Poliak et al., 2002). Notably, the claudin C-terminal domain contains multiple serine, threonine, and tyrosine residues (Hornbeck et al., 2015). Mass spectrometry (MS) data have revealed multiple phosphorylation sites in the C-terminal region of many claudins (Hornbeck et al., 2015; Van Itallie and Anderson, 2018). Although the regulatory role of claudin phosphorylation has not yet been directly studied at the brain microvascular endothelium, studies in other epithelial and endothelial tissues have shown roles in modulation of protein-protein interactions (Nomme et al., 2015), intracellular trafficking (Van Itallie et al., 2012; Twiss et al., 2013), and protein turnover mechanisms (Mandel et al., 2012; Hou et al., 2019). For example, Rho kinase (RhoK) has been demonstrated by Yamamoto et al. (2008) to phosphorylate claudin-5 at tyrosine 207 (T207) in mouse and human brain tissues. This observation was correlated with decreased TEER, suggesting that Rho-associated protein kinase (ROCK) signaling and associated claudin-5 phosphorylation may be a critical modulatory mechanism for TJ permeability at the BBB (Yamamoto et al., 2008).

There are three tight junction-associated marvel proteins (TAMPs) that have been identified at the mammalian BBB. These proteins are known as tricellulin, marvelD3, and occludin. Tricellulin plays a prominent role in regulating paracellular permeability to macromolecules (Krug et al., 2009; Haseloff et al., 2015) while marvelD3 appears to play a compensatory sealing role at the BBB (Raleigh et al., 2010). In contrast, occludin (molecular weight ~65 kDa), the first integral membrane protein identified at BBB TJs has been more extensively characterized. Occludin knockout mice exhibit structurally and functionally intact TJs, possibly due to compensation from other TAMPs; however, these mice display several histological abnormalities and postnatal growth retardation (Saitou et al., 2000). One study suggested that occludin’s physiological role in epithelial barriers is associated with epithelial differentiation rather than establishing barrier properties (Schulzke et al., 2005). Occludin has also been shown to be an important regulator of TJ assembly and functions (Hirase et al., 2001; Van Itallie et al., 2010; Doerfel and Huber, 2012; Buschmann et al., 2013). Additionally, occludin can assemble into higher-order oligomeric assemblies, which are essential to maintenance of TJ functional integrity (McCaffrey et al., 2008). Indeed, our laboratory has shown that disruption of occludin oligomeric structures results in BBB dysfunction characterized by an increase in paracellular permeability (Lochhead et al., 2010, 2012).

Occludin contains a tetraspanning marvel domain, two ECLs, and three cytoplasmic domains, including a short N-terminal domain, a long C-terminal domain, and an intracellular short turn. As has been demonstrated in a study with mutant occludin isoforms in MDCK cells, both ECLs together with at least one transmembrane domain serve important roles in selective permeability of TJs (Balda et al., 2000). The N-terminus of occludin contributes to maintenance of TJ integrity, as evidenced by the observation that deletion of the occludin N-terminal sequence leads to higher paracellular permeability and lower TEER (Bamforth et al., 1999). Lateral (cis-) homodimerization or oligomerization of occludin is mediated by the marvel motif, the cytoplasmic C-terminus, and the second extracellular loop (ECL2; Blasig et al., 2006; Yaffe et al., 2012). These domains are sensitive to changes in redox conditions due to the disulfide bonds between cysteine residues on ECL2 and the occludin C-terminal sequence (Walter et al., 2009; Bellmann et al., 2014). The C-terminus also interacts with ZO proteins (Li et al., 2005; Tash et al., 2012), an interaction that is essential for the trafficking of occludin to the TJ (Furuse et al., 1994). Similar to the claudins, the C-terminal domain of occludin is rich in potential phosphorylation sites and has been shown to interact with a wide range of kinases and phosphatases (Smales et al., 2003; McKenzie et al., 2006; Suzuki et al., 2009; Raleigh et al., 2011; Walsh et al., 2011). With few exceptions, *in vitro* experiments have repeatedly demonstrated that elevated phosphotyrosine and diminished phosphoserine/threonine are generally associated with disruptive barrier functions by interfering with occludin-ZO interactions and/or occludin trafficking (Kale et al., 2003; Elias et al., 2009; Suzuki et al., 2009; Han et al., 2010; Walsh et al., 2011). Interestingly, the phosphorylation status of the C-terminus of occludin is highly dependent on cellular localization of the protein itself; TJ-localized occludin is phosphorylated at a much higher level than those localized to the lateral membrane (Sakakibara et al., 1997), highlighting the importance of examining both cellular expression and localization when studying TJ proteins.

JAMs-1, -2, and -3 are members of the immunoglobulin superfamily that are highly enriched at TJs of the cerebral microvasculature. They are not required for TJ formation (Otani et al., 2019); however, complementary DNA (cDNA) transfection of JAMs in non-TJ forming cell cultures resulted in recruitment of occludin to regions of cell-cell contacts (Bazzoni et al., 2000). Such an observation suggests a potential role for JAMs in mediating occludin trafficking to the TJ. Additionally, loss of JAM protein expression and/or migration of JAM proteins away from the TJ directly lead to loss of BBB properties at the microvascular endothelium (Haarmann et al., 2010; Wang et al., 2014). JAMs consist of a single membrane-spanning domain, a short C-terminal tail, and an immunoglobulin G (IgG)-like extracellular domain. The extracellular domain of JAM proteins mediate homo‐ or hetero-dimerization of JAM family members in both trans‐ and cis-manners, contributing to adherence of adjacent cells at the TJs (Bazzoni et al., 2000). The cytoplasmic C-terminus contains multiple phosphorylation sites and ends with a PDZ binding motif which interacts with ZO-1 as well as other PDZ-containing proteins in the endothelial cells (Bazzoni et al., 2000; Ebnet et al., 2003). Binding affinity of JAMs to ZO-1 may be regulated through the phosphorylation of protein residues on the C-terminal sequence (Van Itallie and Anderson, 2018).

ZO-1 (225 kDa), ZO-2 (160 kDa), and ZO-3 (130 kDa) belong to the PDZ domain-containing protein and membrane-associated granulated kinase (MAGUK) family, which constitute the most prominent intracellular proteins at the TJs. ZO family members are essential for assembly of functional TJs by binding to and cross-linking a wide range of TJ proteins and tethering them to the actin cytoskeleton (Fanning et al., 1998; Shin and Margolis, 2006). The ZO proteins share considerable structural similarity except for the C-terminal sequence that is unique to each specific ZO protein (Bauer et al., 2014). Indeed, differences in these C-terminal protein sequences could explain functional differences of individual ZO family members. While ZO-1 and ZO-2 are considered indispensable for TJ assembly (Umeda et al., 2006), the specific role of ZO-3 at BBB TJs has not been not well-documented. Structurally, the ZO proteins are comprised of three PDZ domains, a Src homology 3 (SH3) domain, a guanylate kinase (GuK) domain, and a proline-rich domain. The first PDZ domain (PDZ1) interacts with the PDZ domain on the C-terminus of most of the claudin proteins (Itoh et al., 1999). The second PDZ domain (PDZ2) mediates ZO-1 heterodimerization with ZO-2 or ZO-3 (Itoh et al., 1999; Wittchen et al., 1999). Structural studies have suggested weak binding between PDZ3 and JAM-1 (Bazzoni et al., 2000). The SH3 domain is the most important element for binding of actin cytoskeleton and signaling molecules (Schmidt et al., 2004; McNeil et al., 2006). Actin cytoskeletal filaments also interact with a defined actin-binding region (ABR) at the C-terminus of the ZO-1 protein (Fanning et al., 2002). It is important to note that there are multiple organizational forms of actin that are regulated by actin-binding proteins which may affect dynamics of TJ proteins and barrier integrity (Tornavaca et al., 2015). GuK are kinases that normally catalyze ATP-dependent transformation of GMP into GDP; however, the GuK domain on the ZO subfamily of MAGUKs is likely enzymatically inactive due to a missing GMP binding-site and steric hindrance at the ATP-binding site (Lye et al., 2010; Zhu et al., 2012). Instead, the GuK domain of ZO proteins has been shown to bind occludin and PDZ motifs of the claudins (Fanning et al., 1998; Chattopadhyay et al., 2014).

## Tight Junction Alterations in Neurological Disorders

**Figure 3:**
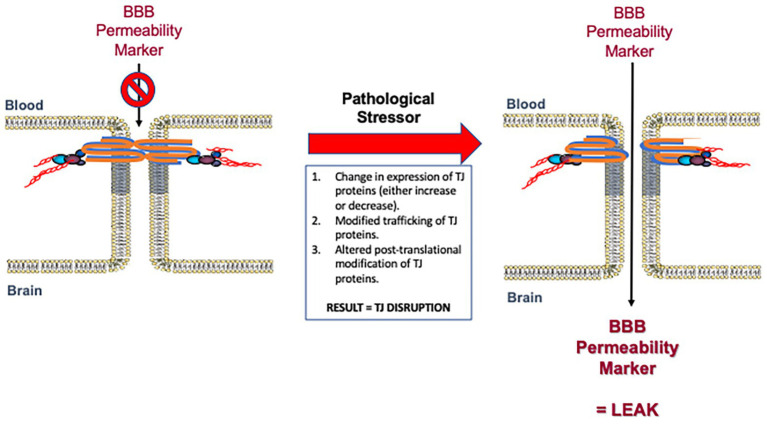
Disruption of blood-brain barrier (BBB) tight junction protein complexes in response to pathological stressors. Pathophysiological mechanisms can cause measurable changes in tight junction functional integrity by various mechanisms. Such mechanisms include (i) modulation in the expression of tight junction proteins as reflected by either an increase or decrease in specific protein levels; (ii) changes in trafficking of constituent proteins away from the tight junction; and/or (iii) altered post-translational modification of specific tight junction proteins. These mechanisms may occur alone or in combination to enable dynamic remodeling of the tight junction in the setting of disease. Changes in the molecular composition of tight junction protein complexes results in increased paracellular permeability (i.e., leak) to specific BBB permeability markers described in Table 1. Modified with permission from Abdullahi et al. (2018).

BBB dysfunction is a pathological feature of several diseases affecting the CNS (Erdo et al., 2017; Reinhold and Rittner, 2017; Costea et al., 2019; Sweeney et al., 2019). BBB permeability alterations are often present before overt clinical symptoms, suggesting that BBB impairment may be a contributing factor to the pathogenesis of various neurological disorders (Figure 3). A number of studies have indicated that plasma proteins can be neurotoxic, which suggests that even if a compromised BBB does not cause these disorders it likely exacerbates them (Chodobski et al., 2011). In this section, we will review the neurological disorders most closely associated with impairments in BBB functional integrity with a focus on human data and *in vivo* studies.

### Alzheimer’s Disease

Alzheimer’s disease (AD) is the most common dementia disorder and is associated with substantial neurovascular dysfunction and pathology (Kisler et al., 2017). Pathological hallmarks of AD include insoluble deposits of amyloid beta (Aβ) protein in the parenchyma and within the walls of blood vessels. Additionally, AD is demarcated by neurofibrillary tangles that are comprised of hyperphosphorylated intraneuronal deposits of the microtubule-associated protein tau (Braak and Del Trecidi, 2015). A number of studies have shown increased extravasation of plasma proteins in AD brains, suggesting dysfunctional BBB properties (Paul et al., 2007; Ryu and McLarnon, 2009; Narayan et al., 2015; Llorens et al., 2019). Several postmortem studies in patients with AD have shown a significant decrease in TJ proteins claudin-5, occludin, and ZO-1 in cerebral blood vessels exhibiting cerebral amyloid angiopathy (Carrano et al., 2011, 2012; Keaney et al., 2015). In contrast, Viggars et al. (2011) showed Alzheimer-type pathology in the cortex that was not associated with downregulation of claudin-5, occludin, or ZO-1 (Viggars et al., 2011). Despite this study, it is now accepted that breakdown of the BBB is an early indicator of cognitive dysfunction and AD (Nation et al., 2019).

Studies in AD animal models display conflicting results regarding BBB permeability and TJ alterations. Tg2576 mice, which overexpress the amyloid precursor protein (APP) with the Swedish mutation (K670N and M671L) showed increased brain uptake of albumin and a fluorescein derivative following transcardial perfusion as well as increased extravasation of IgG and fibrin (Ujiie et al., 2003; Biron et al., 2011; Winkler et al., 2015). Several different laboratories have measured decreased levels of TJ proteins, such as claudin-1, claudin-5, ZO-1, and/or occludin in brain microvessels of Tg2576 mice (Biron et al., 2011; Hartz et al., 2012; Keaney et al., 2015; Winkler et al., 2015). Immunofluorescence staining of ZO-1 and occludin in Tg2576 mice displayed punctate, discontinuous, or interrupted immunoreactivity in the cerebrovasculature, suggesting compromised TJ integrity at the BBB (Biron et al., 2011). Interestingly, knockdown of claudin-5 and occludin at the BBB with small interfering RNA (siRNA) facilitated clearance of Aβ from the brain to the blood, suggesting that targeting TJ function can have beneficial effects in Tg2576 mice (Keaney et al., 2015). Ultrastructural analysis of the brain vasculature in 5XFAD mice [which express mutant human APP with the Swedish mutation, Florida mutation (I716V), and London mutation (V717I), as well as human presenilin-1 harboring two familial AD mutations (M146L and L286V)] showed TJs that were significantly shorter (Kook et al., 2012). In contrast to these findings, Bien-Ly et al. (2015) found no changes in BBB permeability to radiolabeled tracers ranging in size from 86 Da to 150 kDa in TauPS2APP mice, which express the Swedish APP mutation, the German presenilin-2 mutation (N141I), and overexpress tau (Bien-Ly et al., 2015). In these studies, no changes in claudin-5 levels were found in TauPS2APP mice or AD brain lysates. Although TJ proteins or integrity were not analyzed, studies in 3xTG-AD mice (which express the APP Swedish mutation), mutated tau (P301L), and mutated presenilin-1 (M146V) also showed no changes in BBB permeability (Bourasset et al., 2009; Mehta et al., 2013). Discrepancies in observed TJ alterations and BBB permeability in the described studies may be due to the presence and/or severity of cerebral amyloid angiopathy (CAA) in AD cases and the animal models used.

### Epilepsy

A number of BBB impairments have been shown in epilepsy, which exhibits abnormal brain activity leading to seizures (Loscher and Friedman, 2020). IgG extravasation and loss of ZO-1 immunostaining have been observed postmortem in both human brain tissue derived from patients with a positive diagnosis of temporal lobe epilepsy and a rat model of limbic epilepsy. IgG leakage and ZO-1 alterations were observed early after status epilepticus (SE) as well as during latent and chronic periods (Rigau et al., 2007). As demonstrated by van Vliet et al. (2007), albumin immunoreactivity around blood vessels was observed in brain tissue from patients who died during SE (van Vliet et al., 2007). This same study showed increased BBB permeability to albumin in a rat model of SE during the latent and chronic epileptic phases, and these changes were positively correlated to seizure frequency. Hyperosmotic opening of the BBB has also been shown to lead to seizures in animals and humans, suggesting that BBB disruption may lead to progression of epilepsy (Marchi et al., 2007; van Vliet et al., 2007). In a rat model of SE, brain capillaries were more permeable to Texas Red, and seizures were associated with a decrease in the TJ proteins claudin-1, claudin-5, occludin, and ZO-1 at the BBB (Rempe et al., 2018). The same study demonstrated that seizure-induced glutamate release results in cytosolic phospholipase A_2_ activation, which leads to barrier leakage by activating the TJ degrading enzymes matrix metalloproteinase (MMP)-2 and MMP-9.

### Hypoxia/Ischemia

Hypoxia is a central component of cerebral ischemia, which is a restriction in blood supply to the CNS typically caused by a thrombus or embolism. Both hypoxia and ischemia have been shown to exhibit an increase in BBB permeability associated with TJ alterations (Sandoval and Witt, 2008; Prakash and Carmichael, 2015; Lochhead et al., 2017; Abdullahi et al., 2018). This increase in BBB permeability may be either continuous and monophasic or biphasic with an early phase occurring during the first few hours after the onset of hypoxia/ischemia and a later phase, which occurs up to several days after the hypoxic or ischemic insult (Kuroiwa et al., 1985; Witt et al., 2008; Prakash and Carmichael, 2015; Merali et al., 2017). The time course and degree of BBB permeability alterations during ischemia are highly dependent on the species and detection methods as well as the type, degree, and duration of occlusion (Prakash and Carmichael, 2015).

We have shown in female Sprague-Dawley rats that, within 20 min of reoxygenation after an acute hypoxic insult [6% oxygen (O_2_) for 1 h], the permeability of the BBB increases to (^14^C)-sucrose (~340 Da). Of particular note, permeability to Evans blue pre-adsorbed to albumin (~80 kDa) was not altered, which suggests that acute hypoxia leads to BBB permeability changes to small molecules but not macromolecules (Witt et al., 2003; Lochhead et al., 2010). Associated with increased BBB permeability were changes in the localization of the TJ proteins occludin, claudin-5, and ZO-1 as well as disruption of high molecular weight oligomers of occludin (McCaffrey et al., 2009; Lochhead et al., 2010; Willis et al., 2010). These changes could be prevented by administration of the antioxidant tempol or the protein kinase C (PKC) inhibitor chelerythrine chloride, suggesting that generation of reactive oxygen species (ROS) and activation of PKC are involved in altering BBB paracellular permeability during acute hypoxia-reoxygenation stress (Lochhead et al., 2010; Willis et al., 2010). In mice exposed to 8% O_2_ for 48 h, an increase in BBB permeability to sodium fluorescein was associated with a significant reduction in occludin expression and changes in localization of occludin and ZO-1 (Bauer et al., 2010). These changes could be prevented by inhibition of vascular endothelial growth factor (VEGF) or MMP-9.

Disruption of the BBB is a well-established feature of ischemic stroke which contributes to brain injury, and increases the likelihood of hemorrhagic transformation (Sifat et al., 2017; Jiang et al., 2018). While some studies have suggested that extravasation in stroke models is due to an increase in brain endothelial transcytosis through vesicles or non-selective channels (Knowland et al., 2014; Krueger et al., 2015; De Bock et al., 2016), many other studies have demonstrated that increased BBB permeability during ischemia is associated with TJ alterations. Jiao et al. (2011) found that BBB permeability to Evans blue was increased during 3–72 h reperfusion after a 2 h middle cerebral artery occlusion (MCAO) in rats, and these changes were associated with observable gaps in TJ complexes and significant decreases in claudin-5, occludin, and ZO-1 (Jiao et al., 2011). In a permanent MCAO model, there was a biphasic increase in BBB permeability to Evans blue up to 120 h, which correlated with a significant decrease in claudin-5 and occludin (Liu et al., 2018). Recently it was shown that mRNA expression levels of claudin-1, claudin-3, claudin-12, and occludin were significantly decreased, ZO-1 mRNA levels displayed no changes, and claudin-5 mRNA was upregulated in the ipsilateral hemisphere vs. the contralateral hemisphere after 3 h of reperfusion following MCAO (Winkler et al., 2020). This same study found a significant decrease in TJ length and, interestingly, that claudin-3 knockout actually decreased the infarct size and edema formation.

Several different mechanisms have been suggested to contribute to TJ alterations and BBB disruption in experimental stroke models. A number of studies have shown that alterations in BBB integrity during ischemia are due to activation of MMPs, which are capable of degrading TJ and extracellular matrix components. MMP-9 knockout mice displayed significantly reduced Evans blue extravasation and ZO-1 downregulation, but no changes in occludin, in a transient MCAO model (Asahi et al., 2001). After transient MCAO in spontaneously hypertensive rats, an increase in BBB permeability to (^14^C)-sucrose and degradation of claudin-5 and ZO-1 were inhibited by the broad spectrum MMP inhibitor (BB-1101; Yang et al., 2007). Gu et al. (2012) showed that MMP activation was the result of downregulation of caveolin-1 by nitric oxide, and inhibition of nitric oxide synthase by L-NAME could reduce Evans blue leakage and ZO-1 downregulation during transient MCAO in rats (Gu et al., 2012). After photothrombotic ischemia in caveolin-1 knockout mice, Evans blue extravasation and MMP activity are significantly increased while claudin-5, occludin, and ZO-1 expression are significantly decreased (Choi et al., 2016). Progesterone or allopregnanolone has been shown to reduce Evans blue extravasation, MMP-2 and MMP-9 activation, and claudin-5 and occludin downregulation when administered 1 h post-occlusion to rats (Ishrat et al., 2010). Induction of systemic inflammation by peripheral administration of interleukin-1β has been shown to cause sustained BBB disruption, MMP-9 activation, and claudin-5, but not occludin, rearrangement in cerebral vessels after transient MCAO in mice (McColl et al., 2008). Administration of angiopoietin-1 reduced Evans blue extravasation and downregulation of occludin and ZO-1 for up to 1 week, following MCAO in rats (Yu et al., 2012).

### Huntington’s Disease

Huntington’s disease (HD) is a neurodegenerative disorder caused by an autosomal dominant mutation, which results in movement, cognitive, and psychiatric problems. Mutant Huntington protein has been shown to aggregate in the blood vessels of the caudate nucleus in HD patients and in brain tissue from R6/2 mice, a preclinical HD model (Drouin-Ouellet et al., 2015). MRI studies in HD patients showed an increase BBB permeability to Gd-DTPA (~550 Da), and postmortem analysis showed an increase in fibrin deposition in the brain parenchyma associated with a significant reduction in occludin and claudin-5 expression at the BBB (Drouin-Ouellet et al., 2015). Induced pluripotent stem cell-derived brain endothelial cells from HD patients displayed lower TEER values and alterations in claudin-5 localization (Lim et al., 2017). One study in R6/2 mice showed evidence of an increase in BBB permeability through both the transcellular and paracellular pathways, wider intercellular clefts of TJs, and a reduction in occludin and claudin-5 at the BBB (Drouin-Ouellet et al., 2015). Another study showed an age-dependent increase in BBB permeability to fluorescein isothiocyanate (FITC)-albumin and a reduction in claudin-5, occludin, and ZO-1 mRNA and claudin-5 protein levels in the cortex and striatum (Di Pardo et al., 2017).

### Multiple Sclerosis

Multiple sclerosis (MS) is a chronic, progressive CNS disorder, in which the immune system attacks myelinated fibers which causes inflammation, scar tissue, or lesions. BBB disruption during MS is thought to influence oligodendrocyte death, axonal damage, demyelination, and lesion development (Alvarez et al., 2011). Breakdown of the BBB identified by gadolinium-enhanced MRI has been shown to be one of the earliest detectable events in the development of most new MS lesions (Kermode et al., 1990). Vascular TJ morphological alterations and extravasation of serum proteins are frequently observed in areas of MS lesions, but are less common in normal appearing white matter and gray matter (Plumb et al., 2002; Kirk et al., 2003; van Horssen et al., 2007; Wosik et al., 2007). Abnormal tight junctions in both secondary progressive MS and primary progressive MS are most frequently observed in active white matter lesions but also persist in inactive lesions (Leech et al., 2007).

Many studies conducted in experimental autoimmune encephalomyelitis (EAE), an *in vivo* model of demyelinating diseases, have shown TJ alterations that correlate with changes in BBB permeability. Although imperfect, the EAE model does display some similarities in CNS pathologies with MS and, therefore, is commonly used as an animal model to study MS-associated mechanisms. Wolburg et al. (2003) showed that during EAE there was a selective loss of claudin-3, but not claudin-5 or occludin, at the BBB (Wolburg et al., 2003). In contrast to these findings, Errede et al. (2012) found that EAE causes claudin-5 to exhibit a punctate staining pattern early in the disease process while occludin moved away from the TJ and showed diffuse cytoplasmic staining (Errede et al., 2012). Another study in an EAE model observed a relocalization of ZO-1, which preceded overt clinical disease and correlated with sites of inflammatory cell accumulation (Bennett et al., 2010). Wang et al. (2016) found a significant increase in BBB permeability to Evans blue that coincided with a decrease in claudin-5, occludin, and ZO-1 levels in brain homogenates of EAE mice, and these BBB alterations could be ameliorated by resveratrol (Wang et al., 2016). Huang et al. (2017) showed similar alterations in BBB permeability, and claudin-5, occludin, and ZO-1 expression levels in cerebellar white matter could be prevented by a Kv1.3 channel blocker in EAE rats (Huang et al., 2017). Claudin-11 protein expression has also been shown to be significantly downregulated in MS patients and in EAE (Uchida et al., 2019). Differences in TJ alteration data between different groups may be due to differences in the specific EAE model, the time points examined, or the tissue in which the analysis was performed.

### Pain

Pain is an unpleasant sensory and emotional experience associated with actual or potential tissue damage, or described in terms of such damage. Using *in situ* brain perfusion, we have shown in several models of peripheral inflammatory pain (PIP) that brain uptake increases to (^14^C)-sucrose, but not to Evans blue-albumin, suggesting that inflammatory pain alters BBB paracellular permeability to small molecules but not macromolecules (Huber et al., 2001; Lochhead et al., 2012). These changes are biphasic in nature and occur within the first 6 h and again at 48 in the hind paw λ-carrageenan model of PIP (Huber et al., 2002). The early phase of these alterations could be prevented or reversed by administration of the non-steroidal anti-inflammatory diclofenac, transforming growth factor-β (TGF-β), tempol, or perineural injection of bupivacaine into the right hind leg, suggesting a role for cyclooxygenase, TGF-β signaling, ROS generation, and nociceptive input in PIP-induced BBB disruption (Brooks et al., 2008; Campos et al., 2008; Ronaldson et al., 2009; Lochhead et al., 2012). Associated with the increase in BBB permeability during PIP are alterations in the expressions of claudin-3, claudin-5, occludin, and ZO-1 as well as disruption of high molecular weight occludin oligomers (Huber et al., 2002; Brooks et al., 2005, 2008; McCaffrey et al., 2008; Ronaldson et al., 2009; Lochhead et al., 2012). At 3 h after induction of PIP, we have observed ROS-mediated disruption of occludin oligomeric assemblies and altered trafficking of occludin (McCaffrey et al., 2008; Lochhead et al., 2012).

In addition to PIP, BBB paracellular permeability has been observed to increase during familial hemiplegic migraine and in animal models of migraine (Gursoy-Ozdemir et al., 2004; Dreier et al., 2005; Olah et al., 2013). In a model of cortical spreading depression (CSD), Evans blue and plasma protein extravasation was associated with an increase in MMP-9 expression and activity and a decrease in ZO-1 expression in brain microvessels (Gursoy-Ozdemir et al., 2004). These changes could be prevented by the MMP inhibitor GM6001 within 3–24 h. In another study of CSD, brain uptake of (^14^C)-sucrose was observed beginning at 30 min with no changes in claudin-5 or occludin expression (Cottier et al., 2018).

### Parkinson’s Disease

Parkinson’s disease (PD) is a neurodegenerative disorder, which affects movement. Evidence of BBB disruption in PD includes the findings of significant increase in albumin and IgG in the cerebrospinal fluid of PD patients (Pisani et al., 2012) and an increase in extravasation of erythrocytes, hemoglobin, and fibrin in the striatum of PD patients (Gray and Woulfe, 2015). Immunofluorecence staining of ZO-1 and occludin is also significantly reduced in the substantia nigra of PD patients, and these findings correlate with an increase in parenchymal IgG (Pienaar et al., 2015). Interestingly, these changes were attenuated in patients subjected to deep brain stimulation. The 6-hydroxydopamine (6-OHDA) preclinical model of PD showed BBB leakage in the substantia nigra and striatum 10 and 34 days after injection of 6-OHDA into the striatum (Carvey et al., 2005). Also observed in the 6-OHDA model was a significant reduction in the TJ proteins ZO-1 and claudin-5 (Huang et al., 2016). In the 1-methyl-4-phenyl-1,2,3,6-tetrahydropyridine (MPTP) mouse model of PD, an increase in BBB permeability to Evans blue and FITC-albumin was observed in the striatum, but not the hippocampus (Chen et al., 2008). These changes correlated with significant decreases of occludin and ZO-1, and could be prevented by pre-administration of caffeine. Other studies in the MPTP model have shown that decreased levels of occludin or ZO-1 could be attenuated by acetyl-L-carnitine or sodium butyrate (Liu et al., 2017; Burks et al., 2019).

### Traumatic Brain Injury

Traumatic brain injury (TBI) is a complex, dynamic, and heterogenous pathology which can be classified as impact or non-impact, focal or diffuse, mild, moderate, or severe. A compromised BBB is a well-established pathological feature of TBI (Saw et al., 2014; Alluri et al., 2015; Prakash and Carmichael, 2015; Rodriguez-Grande et al., 2017). Intracranial hemorrhage represents the most serious complication of TBI and can be detected in moderate to severe cases (Narayan et al., 2008; Currie et al., 2016). In pre-clinical models of TBI, BBB disruption can be monophasic or biphasic in nature and can occur within minutes to days after injury similar to what is observed in ischemic stroke models (Shapira et al., 1993; Baldwin et al., 1996; Barzo et al., 1996). Interestingly, in the mouse cerebral contusion model, BBB permeability to horseradish peroxidase (44 kDa) was seen up to 5 h post-injury while permeability to smaller molecules (~0.3–10 kDa) can be measured up to 4 days, suggesting that TBI can cause size-selective BBB leakage (Habgood et al., 2007). TJ proteins such as claudin-5, occludin, and ZO-1 are downregulated after the first few days of TBI but can be upregulated 1–2 weeks after injury when the BBB regains functional integrity (Lin et al., 2010; Wen et al., 2014). In addition to paracellular alterations, transcellular permeability across the BBB may also be increased after TBI (Castejon, 1984; Nag et al., 2007). Of particular note, Ren et al. (2013) showed increased Evan’s blue albumin extravasation at 24 h following moderate closed skull TBI in male C57Bl/6 mice, suggesting that even less severe trauma can lead to measurement BBB disruption (Ren et al., 2013). A number of different mechanisms have been suggested to contribute to BBB dysfunction during TBI including TGF-β signaling, glutamate excitotoxicity, generation of ROS, activation of MMPs, inflammation, and VEGF signaling (Chodobski et al., 2011).

## Modulation of BBB Permeability to Enhance Drug Delivery

Although the BBB plays an important protective role of the CNS, modulation of TJs may allow increased brain delivery of drugs through the paracellular route (Deli, 2009; Tscheik et al., 2013; Greene and Campbell, 2016). For example, we have shown that TJ alterations during PIP are associated with improved CNS delivery of the opioid analgesic codeine, which results in enhanced analgesia (Hau et al., 2004). Ideally, TJ alterations at the BBB would be transient in nature to allow enhanced drug permeation into the CNS without causing side effects or toxicity resulting from an open barrier. Pharmacological strategies to modulate TJs at the BBB must also exhibit low systemic toxicity as their delivery to brain endothelial cells would likely be through the bloodstream. Over the past several years, a number of different strategies have been tested to increase BBB paracellular permeability with the goal of improving drug delivery to the brain *in vivo*.

One strategy to increase brain delivery of drugs is intracarotid infusion of hyperosmotic concentrations of arabinose or mannitol. This technique causes reversible vasodilation and shrinkage of endothelial cells, which leads to a loosening of the TJs, an increase in paracellular diffusion, and bulk fluid flow across the BBB (Rapoport and Robinson, 1986; Rapoport, 2000; Dobrogowska and Vorbrodt, 2004). While this method has been used to enhance delivery of chemotherapeutics to brain tumors, limited efficacy and severe side effects such as brain edema and seizures have prevented its widespread use in the clinic (Rapoport, 2000; Marchi et al., 2007). In addition, its invasive nature and need for patients to be anesthetized during the procedure render the technique impractical for chronic administration of therapeutics (Marchi et al., 2007; Hersh et al., 2016).

Several different attempts to pharmacologically manipulate BBB permeability to increase brain delivery of drugs have also been attempted. BBB permeability to Evans blue was reversibly increased 15 min after intracarotid infusion of oleic acid in rats (Sztriha and Betz, 1991). Administration of oleic acid or linoleic acid to cats also caused BBB disruption as measured by MRI, but these effects were associated with brain edema, necrosis, and demyelination (Kim et al., 2005). Intravenous infusion of the bradykinin B_2_ receptor agonist RMP-7 (Cereport®) increased lanthanum penetration through TJs of the BBB by loosening the junctional complexes (Sanovich et al., 1995). RMP-7 also showed enhanced uptake of chemotherapeutics in preclinical brain tumor models but was ineffective when used to enhance brain delivery of carboplatin to treat childhood brain tumors in a phase II trial (Emerich et al., 2001; Warren et al., 2006). Administration of alkylglycerols has also been shown to increase BBB permeability of a number of different drugs and tracers (Iannitti and Palmieri, 2010). Intracarotid administration of alkylglycerols induce a size-dependent permeability increase, suggesting that alkylglycerols increase BBB paracellular permeability by modulating TJs, but intracarotid administration is an invasive procedure, and alkylglycerol administration to increase CNS drug uptake has not been tested clinically (Erdlenbruch et al., 2003). A 12 kDa fragment of the ZO toxin (Δ*G*) from *vibrio cholerae* has been shown to increase BBB to (^14^C)-sucrose, likely by activating PKC (Fasano et al., 1995; Menon et al., 2005). These effects were modest without the addition of protease inhibitors, suggesting that Δ*G* is rapidly degraded in the bloodstream and likely not a good candidate to clinically improve drug delivery to the CNS (Menon et al., 2005).

Another strategy to transiently increase BBB paracellular permeability is by targeting specific components of the TJs with siRNA or novel peptidomimetic drugs. Campbell et al. (2008) showed that BBB paracellular permeability could be significantly increased up to 48 h after tail vein injection of siRNA targeting claudin-5 in mice (Campbell et al., 2008). This technique led to enhanced brain delivery of Gd-DTPA (742 Da), but not to FITC-dextran (4.4 kDa), showing a size-selective barrier alteration when targeting claudin-5 similar to what is seen in claudin-5 knockout mice. More recently, Dithmer et al. (2017) reported that newly engineered peptidomimetic drugs targeting claudin-5 could increase paracellular BBB permeability to various tracers across a wide molecular weight range such as lucifer yellow (457 Da), FITC-dextran (10 and 40 kDa), FITC-albumin (67 kDa), and tetramethylrhodamine-dextran (155 kDa) (Dithmer et al., 2017). These claudin-5 targeting peptides were also shown to increase CNS distribution of gadolinium 4 h following their administration, suggesting a novel approach for transient and reversal opening of the BBB for optimization of drug delivery (Dithmer et al., 2017).

Ultrasound in the presence of microbubbles is a technique that is increasingly being used to create a transient and focal opening of the BBB to deliver therapeutics to the CNS ranging in size from small molecules to antibodies, plasmids, and viral vectors. Focused ultrasound (FUS) bursts generated from an external source are able to utilize microbubbles as contrast agents that act as cavitation sites, which physically disrupt the BBB (Alonso, 2015; McMahon et al., 2019). Increased BBB permeability induced by ultrasound may involve transcytosis, opening of transendothelial channels, passage through injured endothelial cells, or opening of TJs (Sheikov et al., 2004). Studies in rats showed that FUS with microbubbles could increase BBB permeability to horseradish peroxidase and lanthanum. This BBB disruption was associated with ultrastructural alterations of claudin-5, occludin, and ZO-1, suggesting that FUS can increase BBB paracellular permeability by altering TJ proteins (Sheikov et al., 2008). Clinical trials utilizing this technique to improve CNS drug delivery are currently underway.

## Conclusions

TJ protein complexes are the major determinants of paracellular permeability at the BBB. While brain microvascular endothelial TJs function to protect the CNS from potentially harmful substances in the blood, their protective role also represents a major obstacle to effectively deliver therapeutics for treatment of neurological diseases. Evidence is increasingly emerging that pathological alterations of TJs at the BBB may exacerbate or possibly initiate neurological dysfunction. A better understanding of how TJ expression, localization, and function is altered at the BBB during disease states may provide opportunities to protect the CNS from adverse consequences resulting from BBB disruption. Conversely, understanding the mechanisms involved in rapidly and transiently regulating TJ function at the BBB may allow improved delivery of therapeutics to the CNS through the paracellular route. Future research elucidating signaling pathways affecting TJ function at the BBB has the potential to both protect the CNS from a compromised barrier and more effectively treat neurological disorders by optimizing drug delivery.

## Author Contributions

JL and JY wrote the paper. PR and JL created the figures and tables. PR and TD edited the paper. All authors contributed to the article and approved the submitted version.

### Conflict of Interest

The authors declare that the research was conducted in the absence of any commercial or financial relationships that could be construed as a potential conflict of interest.
