# Supplementary material for: Structure, Function, and Regulation of the Blood-Brain Barrier Tight Junction in Central Nervous System Disorders
Source: Front Physiol. 2020 Aug 6;11:914. doi: 10.3389/fphys.2020.00914 (PMC7424030; doi:10.3389/fphys.2020.00914)
Supplement: Supplementary file 1 [file Data_Sheet_1.DOCX]

**Table 1:** **Common BBB Permeability Markers used for Measurement of Paracellular Leak**

| **Tracer** | **Molecular Weight (kDa)** | **Comments** | **References** |
| --- | --- | --- | --- |
| Sucrose | 0.3423 | - Polar vascular marker that is not transported and does not cross the intact BBB. - Useful for measurement of transient or subtle BBB leak. - Typically radiolabeled to allow for detection in brain tissue or brain fluids. | (Bhattacharjee et al., 2001; Saunders et al., 2015) |
| Sodium Fluorescein (NaF) | 0.3763 | - Small molecular weight marker commonly used to assess BBB paracellular permeability. - Weakly binds to plasma proteins. - Transport substrate for organic anion transporter 3 (OAT3) and multidrug resistance protein 2 (MRP2). Since both proteins transport substrates from brain to blood, use of NaF may lead to underestimations in BBB leak. | (Wolman et al., 1981; Hawkins and Egleton, 2006; Kaya and Ahishali, 2011; Saunders et al., 2015) |
| Lucifer Yellow | 0.4423 | - Small molecular weight marker for assessment of BBB integrity. - Can only access brain tissue via passive paraceullar diffusion. - Useful for measurement of transient or subtle BBB leak. | (Omidi et al., 2003; Qosa et al., 2016) |
| Evans blue | 0.9608 | - Small molecular weight dye that is commonly used to assess BBB dysfunction in *in vivo* model system. - Depending on the concentration used, a percentage will bind to plasma proteins such as albumin and a percentage will remain free. - Due to perceived binding to albumin, Evan’s blue staining in brain tissue is believed to reflect large-scale BBB opening; however, the fact that some dye remains unbound to plasma proteins makes it impossible to use Evan’s blue as a reflection of size selectivity of BBB leak. | (Kaya and Ahishali, 2011; Saunders et al., 2015) |
| Dextrans | 3 – 2,000 | - Available across a wide range of molecular weights, which enables assessment of size-dependent BBB leak. - Can be conjugated to fluorescent labels (i.e., FITC, tetramethylrhodamie) or to biotin to enable detection in brain tissue or brain fluids. - Relatively inert and resistant to metabolic enzymes. | (Willis et al., 2010; Saunders et al., 2015; Natarajan et al., 2017; Devraj et al., 2018) |
| Horseradish Peroxidase (HRP) | 44 | - Allows for assessment of BBB permeability to macromolecules. - Reaction product can be made electron-dense, thereby enabling visualization by electron microscopy. - Can cause mast cell degranulation, which can affect BBB permeability in *in vivo* experiments. Therefore, HRP may overestimate BBB leak under these conditions. | (Kaya & Ahishali, 2011;  Saunders et al. 2015) |
| Albumin | 66.5 | - Plasma protein that does not cross the intact BBB. - Useful for evaluated large-scale BBB disruption. - Can be fluorescently or radio-labeled to allow for measurement of duration or progression of BBB leak. | (Saunders et al. 2015) |
| IgG | 150 | - Large molecular weight tracer that enables assessment of large-scale BBB disruption. - Can be fluorescently or radio-labeled to allow for measurement of duration or progression of BBB leak | (Saunders et al. 2015) |
| Fibrinogen | 340 | - Large molecular weight tracer that enables assessment of large-scale BBB disruption. - Can be fluorescently labeled to allow for measurement of duration or progression of BBB leak | (Saunders et al. 2015) |
